# Research skills for undergraduates: a must!

**DOI:** 10.1007/s40037-013-0054-3

**Published:** 2013-04-10

**Authors:** Thomas I. Lemon, Rhianon Lampard, Benjamin A. Stone

**Affiliations:** c/o 5th floor Cochrane building, University Hospital of Wales, Cardiff, CF14 4XW UK

Dear Sirs,

Research serves a vital purpose in furthering the knowledge base on which medicine is practised. Furthermore it may be defined as the method to which we *inform our action*. In order for us to practice evidence-based medicine, we must be informing our action, which is reliant on robust and rigorous research.

Tomorrow’s Doctors 2009 relates to three domains: the scholar, the scientist and the practitioner. All three domains are intrinsically linked and responsible for consistently increasing the knowledge base [1] and hence require consistent, thorough and accurate research [2, 3]. Thus it is only logical that the authors of Tomorrow’s Doctors, the General Medical Council, expect these graduates to have the skills to perform this task.

Anecdotal evidence, however, suggests research is often seen by medical students as something only students who intercalate or come from previous academic backgrounds can achieve. Research techniques and methodologies are practically absent from the busy curriculum facing medical undergraduates. Furthermore, anecdotal evidence and conference presentations suggest many students do not feel they have any research skills teaching throughout their undergraduate course. The absence of research skills in the general undergraduate curriculum therefore jeopardizes the furthering of each domain, but also the ability to practice current and relevant evidence-based medicine.

Some institutions have tried to address this issue, in part by attempting to inform medical students about the benefits of careers in clinical research and academic medicine. However, this is doomed to failure if the people they wish to fill these positions in the future are graduating from courses that have not given them any skills to firstly partake in research, but more importantly to have an insight into what a research career may be like, compared with a clinical career.

Whilst no one should be forced to carry out research, as this may encourage a propensity for ‘bad research’ to flood academia, the basic concepts required to construct a research paper, and understand when and where ethics is and is not required should be present on undergraduate curriculums. The authors would argue research skills are an imperative core inclusion to the undergraduate curriculum, in order to ensure research uptake at some level in the majority of Tomorrow’s Doctors further practice.
